# S1P Stimulates Erythropoietin Production in Mouse Renal Interstitial Fibroblasts by S1P_1_ and S1P_3_ Receptor Activation and HIF-2α Stabilization

**DOI:** 10.3390/ijms22179467

**Published:** 2021-08-31

**Authors:** Redona Hafizi, Faik Imeri, Roland H. Wenger, Andrea Huwiler

**Affiliations:** 1 Institute of Pharmacology, University of Bern, Inselspital, INO-F, CH-3010 Bern, Switzerland; redona.hafizi@pki.unibe.ch (R.H.); faik.imeri@pki.unibe.ch (F.I.); 2 Institute of Physiology, University of Zürich, CH-8057 Zürich, Switzerland; roland.wenger@access.uzh.ch

**Keywords:** erythropoietin, sphingosine 1-phosphate, S1P receptors, fingolimod, hypoxia, renal interstitial fibroblasts, protein kinase C

## Abstract

Erythropoietin (Epo) is the critical hormone for erythropoiesis. In adults, Epo is mainly produced by a subset of interstitial fibroblasts in the kidney, with minor amounts being produced in the liver and the brain. In this study, we used the immortalized renal interstitial fibroblast cell line FAIK F3-5 to investigate the ability of the bioactive sphingolipid sphingosine 1-phosphate (S1P) to stimulate Epo production and to reveal the mechanism involved. Stimulation of cells with exogenous S1P under normoxic conditions (21% O_2_) led to a dose-dependent increase in Epo mRNA and protein levels and subsequent release of Epo into the medium. S1P also enhanced the stabilization of HIF-2α, a key transcription factor for Epo expression. S1P-stimulated Epo mRNA and protein expression was abolished by HIF-2α mRNA knockdown or by the HIF-2 inhibitor compound 2. Furthermore, the approved S1P receptor modulator FTY720, and its active form FTY720-phosphate, both exerted a similar effect on Epo expression as S1P. The effect of S1P on Epo was antagonized by the selective S1P_1_ and S1P_3_ antagonists NIBR-0213 and TY-52156, but not by the S1P_2_ antagonist JTE-013. Moreover, inhibitors of the classical MAPK/ERK, the p38-MAPK, and inhibitors of protein kinase (PK) C and D all blocked the effect of S1P on Epo expression. Finally, the S1P and FTY720 effects were recapitulated in the Epo-producing human neuroblastoma cell line Kelly, suggesting that S1P receptor-dependent Epo synthesis is of general relevance and not species-specific. In summary, these data suggest that, in renal interstitial fibroblasts, which are the primary source of plasma Epo, S1P_1 and 3_ receptor activation upregulates Epo under normoxic conditions. This may have a therapeutic impact on disease situations such as chronic kidney disease, where Epo production is impaired, causing anemia, but it may also have therapeutic value as Epo can mediate additional tissue-protective effects in various organs.

## 1. Introduction

The prevalence of chronic kidney disease (CKD) is constantly increasing worldwide. In 2019, CKD affected 15% of the U.S. population [1]. This number is strongly influenced by the increasing incidence of risk factors, such as hypertension and diabetes, resulting in CKD as a major public health concern. Renal fibrosis is a hallmark of many forms of CKD. Tissue fibrosis is the consequence of continuous chronic inflammation and immune response in the kidney, leading to dysregulation of repair processes, tissue remodelling, and extracellular matrix deposition, which cumulates in the progressive loss of kidney function and finally end-stage renal disease and renal failure [2,3].

As kidney disease progresses, a major complication that develops is anemia, affecting nearly all patients in the late stages of CKD. Anemia is associated with a reduced quality of life and increased cardiovascular disease, hospitalization, and mortality [4]. The reason for this complication is an inadequate production of erythropoietin (Epo), which is the main hormone driving erythropoiesis, regulating the oxygen-load of the blood [5,6,7,8]. Additionally, Epo exerts tissue-protective effects in various organs, including the heart, kidney, brain, liver, and colon [9,10,11,12,13,14]. In the kidney, Epo protects from hemorrhagic shock- and ischemia/reperfusion-induced injury [10,15,16,17], sepsis-induced acute injury [18], and diabetes-induced injury [19]. While the erythrogenic effect of Epo is mediated by binding to an (EpoR)_2_ homodimeric complex, the protective effect of Epo evolves through binding and activation of an EpoR/β-common receptor (βcR) heterodimeric complex [20]. In adults, the primary source of plasma Epo, contributing to 90% of circulating Epo, is the kidney, where peritubular cells in the renal cortex mainly fulfill this task [21]. The remaining 10% is produced in the liver and a minor part in the brain [8,22]. Epo production is mainly regulated on the mRNA level, and the key transcription factor involved is the hypoxia-inducible factor (HIF)-2α [23]. Therefore, systemic hypoxia, i.e., reduced oxygen levels in the blood owing to anemia or hypoxaemia, is a classical inducer of renal Epo production [24]. Other stimuli have also been described, including testosterone, somatotropin, insulin-like growth factor 1, thyroid hormone, and retinoic acid [25,26,27].

Sphingolipids represent a main class of cellular lipids. They have mainly structural functions, but some subspecies are now appreciated as bioactive molecules and have signalling properties regulating physiological and pathophysiological functions in many organ systems including the kidney [28,29,30]. Sphingosine 1-phosphate (S1P) is one of these active subspecies. S1P is synthesized intracellularly by the two sphingosine kinases (Sphk) 1 and 2 [31,32], and can act intracellularly as a second messenger to exert cellular responses, or extracellularly as a ligand of a family of G protein-coupled receptors (GPCRs), denoted S1P_1-5_. When activated by S1P, these receptors couple to a variety of signalling cascades, including the mitogen-activated protein kinase (MAPK/ERK), the protein kinase (PK) B/Akt, and PKC. As a result, S1P influences the migration, proliferation, survival, inflammatory mediator, and extracellular matrix production of cells [28,33,34,35,36].

Several sphingolipids subspecies, including S1P, were identified in the kidney to accumulate in various forms of CKD that typically end in renal fibrosis [28,30]. S1P was shown to have both pro-fibrotic and anti-fibrotic potential, depending on its site of production [33]. The two S1P-generating enzymes, Sphk1 and Sphk2, seem to have opposite effects in mouse models of CKD, such as in diabetes-induced glomerulosclerosis and in unilateral ureteral obstruction (UUO)-induced tubulointerstitial fibrosis. While the depletion or inhibition of Sphk1 aggravated disease symptoms, depletion or inhibition of Sphk2 rather reduced disease symptoms [37,38,39,40,41,42,43]. Based on these data, it is tempting to speculate that the subcellular site of S1P production and/or action causes the difference in disease outcome.

A link between hypoxia and S1P was shown early on. Hypoxia exposure of vascular smooth muscle cells in culture led to an increased cellular S1P production and cell growth, and both effects were abolished by an Sphk inhibitor [44]. Hypoxia also upregulated Sphk1 mRNA expression in endothelial cells [45] and cancer cells [46], and this was mediated by the transcription factors HIF-1α and HIF-2α, which can bind to one of the two hypoxia response elements (HREs) identified in the human Sphk1 promoter region [45]. On the other side, an opposite signal flow from S1P to HIF-1/2 was also reported. Through S1P receptor activation, S1P could stabilize HIF-1α and HIF-2α in various cancer cell lines [47,48,49,50] including renal clear cell carcinomas [51]. This implies a potential effect of S1P on a multitude of existing HIF target genes, including Epo. However, this putative link between S1P and renal Epo production has still not been addressed. A main obstacle has been that the renal Epo-producing (REP) cells, which are considered to be renal interstitial fibroblasts, have not been available until recently, when we isolated and immortalized REP cells [52,53]. Selected clones were characterized as Epo-producing fibroblast-like cells and named fibroblastoid atypical interstitial kidney (FAIK) cells [52].

In this study, we have used the FAIK F3-5 cell line and demonstrate for the first time that extracellular S1P, and its analogue FTY720, stimulate increased Epo protein synthesis and secretion under normoxic conditions. This effect is mechanistically due to activation of S1P_1_ and S1P_3_ receptors and involves HIF-2α protein stabilization. Furthermore, we show that the S1P-dependent stimulation of Epo involves the classical- and stress-activated protein kinases ERK and p38-MAPK, as well as PKC and PKD activation. A similar effect of S1P is found in the human neuroblastoma cell line Kelly, suggesting a species-independent and general effect of S1P on Epo-producing cells. In summary, these data suggest that S1P promotes Epo production, which could have a therapeutic impact for the treatment of anemia in patients with CKD.

## 2. Results 

**Figure 1:**
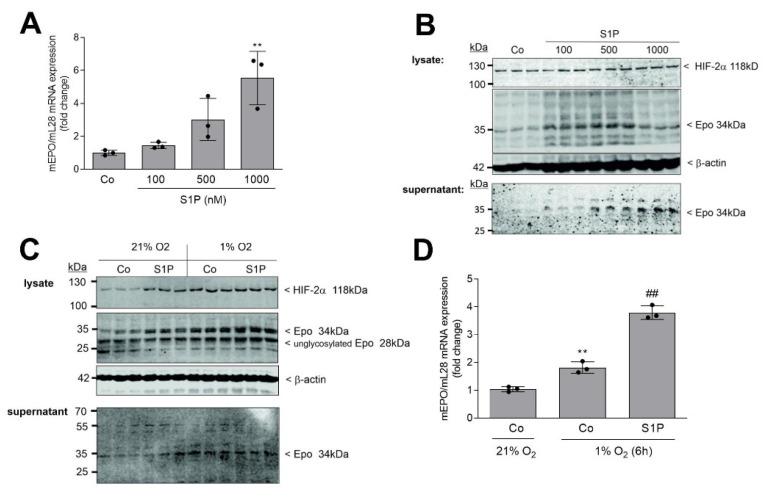
Effect of S1P and hypoxia on Epo mRNA and protein expression in F3-5 cells. (**A**,**B**): Confluent F3-5 cells were incubated for 16 h with serum-free DMEM prior to stimulation for 6 h with the indicated concentrations of S1P. (**C**,**D**): Cells were incubated for 6 h under normoxia (21% O_2_) or hypoxia (1% O_2_) in the absence (Co) or presence of 500 nM S1P. RNA extracts (**A**,**D**) were prepared and taken for quantitative PCR analysis using primers for mEpo and mL-28 (for normalization). ΔΔCt values were calculated and the results show the fold change compared with the untreated control and are means ± S.D. (*n* = 3), ** *p* < 0.01 considered statistically significant when compared with the control samples; ^##^ *p* < 0.01 compared with the 1% O_2_-Co samples. Protein extracts (**B**,**C**) were prepared and taken for protein separation by SDS-PAGE; transferred to nitrocellulose membranes; and subjected to Western blot analyses using antibodies against mouse Epo, HIF-2α, and β-actin. Bands in B and C, corresponding to Epo, HIF-2α, and β-actin, were evaluated by Image Studio Lite software, and results are depicted in Supplementary Figure S1.

In this study, we aimed to investigate the impact of S1P receptor activation on renal Epo production. Therefore, we used the previously isolated and characterized mouse REP cell line FAIK F3-5 [52]. These cells were stimulated for 6 h with increasing concentrations of exogenous S1P and samples were analyzed for Epo mRNA and protein expression. As seen in Figure 1A, S1P stimulated a concentration-dependent increase of Epo mRNA with a maximal effect at 1 μM S1P.

The protein expression of Epo in cellular lysates was detected by Western blot analysis. Epo is a heavily glycosylated and sialylated protein, hence various bands are expected to be detected. The primary and most active form of Epo runs at 34 kDa [54], which was increased by S1P stimulation, showing the highest effect at 500 nM (Figure 1B). As Epo is a secreted protein, we also analyzed the supernatant of stimulated cells. As expected, S1P also enhanced the secreted forms of Epo (Figure 1B, lower panel). Exposure of cells to hypoxia of 1% O_2_ also increased Epo protein expression (Figure 1C), confirming our previous data [52]. Co-treatment of S1P under 1% O_2_ showed an additive effect on Epo mRNA expression (Figure 1D), while the protein was only slightly further enhanced by co-treatment (Figure 1C). In the same experimental setting, we also detected the HIF-2α transcription factor, which is known to be the key regulator of Epo in the kidney [55,56], as well as in interstitial fibroblasts [57] and FAIK 3-5 cells [52]. S1P treatment resulted in a concentration-dependent stabilization, and thus increased protein expression of HIF-2α (Figure 1B, top panel).

**Figure 2:**
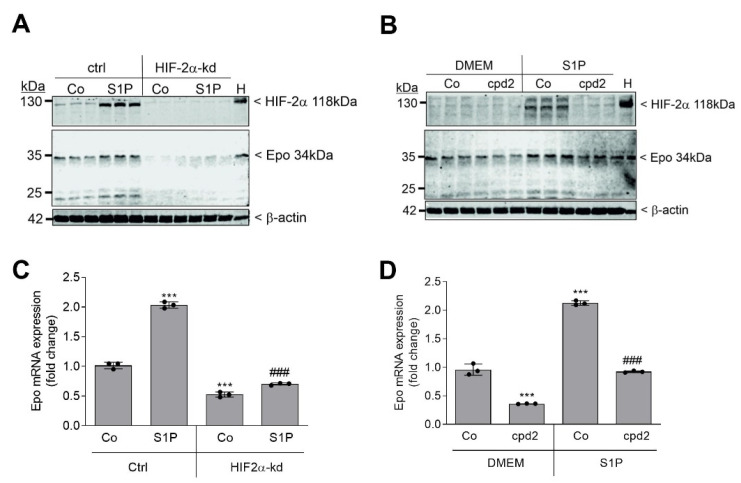
Effect of HIF-2α knockdown and an HIF-2α inhibitor on S1P-stimulated Epo protein and mRNA expression in F3-5 cells. (**A**,**C**) Confluent Ctrl or HIF-2α knockdown (kd) cells were serum-starved for 16 h prior to stimulation for further 6 h with either vehicle (Co) or S1P (500 nM). (**B**,**D**) Cells were pretreated for 30 min with the HIF-2α inhibitor compound 2 (cpd2) at 10 μM prior to stimulation for 6 h with vehicle (Co) or S1P (500 nM) in the presence of cpd2. As positive control, lysates of the neuroblastoma cell line Kelly, exposed for 16 h to 1% O_2_, were used (H). Thereafter, protein extracts (**A**,**B**) or RNA extracts (**C**,**D**) were prepared and proteins were separated by SDS-PAGE; transferred to nitrocellulose membranes; and subjected to Western blot analyses using antibodies against mouse Epo, HIF-2α, or β-actin. RNA was taken for quantitative PCR analysis using primers of mouse Epo and mL28 for normalization. ΔΔCt values were calculated as fold increase compared with the untreated control and means ± S.D. (*n* = 3), *** *p* < 0.001 was considered statistically significant when compared with the control samples. ^###^ *p* < 0.001 compared with the S1P-stimulated control samples. Bands corresponding to Epo and β-actin were evaluated by Image Studio Lite software and the results are depicted in Supplementary Figure S2.

To determine the impact of HIF-2α on S1P-stimulated Epo, we stably depleted cells of HIF-2α by lentiviral transduction of an shRNA construct [52]. Stimulating these cells with S1P failed to increase Epo protein, demonstrating that the S1P effect on Epo is fully dependent on HIF-2α (Figure 2A).

In an alternative approach, F3-5 cells were treated with an HIF-2α inhibitor, denoted as compound 2, which acts as an allosteric inhibitor of HIF-2 by antagonizing HIF-2 heterodimerization with HIF-β [58]. Again, S1P-stimulated HIF-2α and Epo expression was strongly reduced (Figure 2B). A similar reduction by both approaches was seen on the mRNA level of Epo (Figure 2C,D).

**Figure 3:**
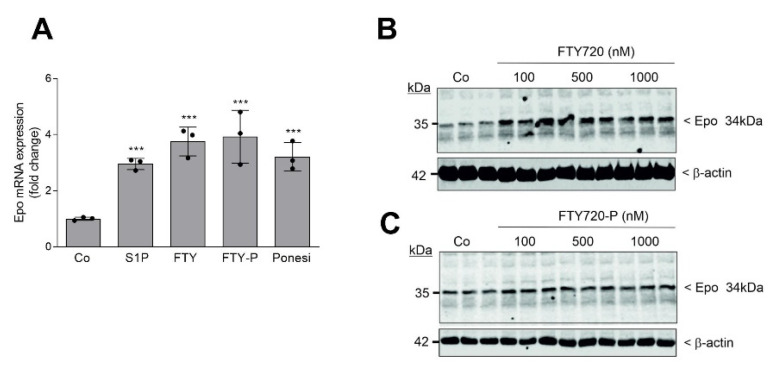
Effect of FTY720 and FTY720-phosphate on mouse Epo mRNA and protein expression in F3-5 cells. (**A**) Confluent and quiescent F3-5 cells were stimulation for 6 h with either vehicle (Co) or 1 μM of S1P, FTY720 (FTY), FTY720-phosphate (FTY-P), and ponesimod (Ponesi). RNA extracts were prepared and taken for quantitative PCR analysis using primers of mouse Epo, and mL28 for equalization. ΔΔCt values were calculated as fold change compared with the control and are means ± S.D. (*n* = 3), *** *p* < 0.001 was considered statistically significant when compared with the control. (**B**,**C**) Quiescent cells were stimulated for 6 h with either vehicle (Co) or the indicated concentrations of FTY720 (**B**) and FTY720-P (C). Thereafter, protein extracts were prepared and separated by SDS-PAGE, transferred to nitrocellulose membranes, and subjected to Western blot analyses using antibodies against mouse Epo and β-actin. Bands corresponding to Epo and β-actin were evaluated by Image Studio Lite software and the results are depicted in Supplementary Figure S3.

We further investigated whether the approved drug fingolimod (FTY720), a S1P receptor modulator that acts as an unspecific agonist of four out of the five S1P receptors, i.e., S1P_1,3,4, and 5_, can mimic the S1P effect on Epo. F3-5 cells were stimulated for 6 h with the same concentration (1 μM) of either S1P, FTY720, FTY720-phosphate, or the S1P_1_-selective agonist ponesimod. All compounds upregulated Epo mRNA expression to a similar extent (Figure 3A). The effects of FTY720 and FTY720-phosphate also occurred in a concentration-dependent manner on the protein levels (Figure 3B,C). 

**Figure 4:**
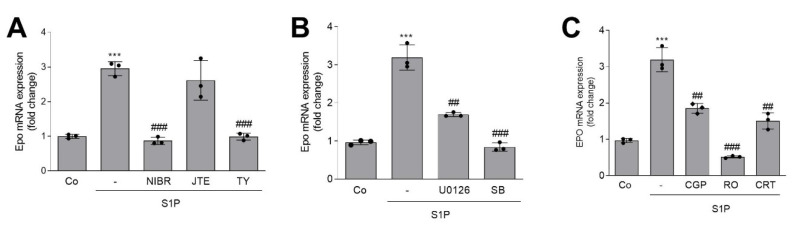
Effect of S1PR antagonists and specific inhibitors of ERK, p38-MAPK, PKC, and PKD on S1P-stimulated Epo mRNA expression in F3-5 cells. Confluent cells were incubated for 16 h in serum-free DMEM prior to stimulation for 6 h with S1P (500 nM) in the absence or presence of the following compounds: NIBR-0213 (**A**, NIBR, 10 μM), JTE-013 (**A**, JTE, 10 μM), TY52156 (**A**, TY, 10 μM), U0126 (**B**, 10 μM), SB203580 (**B**, SB, 10 μM), CGP41251 (**C**, CGP, 100 nM), RO-318220 (**C**, RO, 1 μM), and CRT0066101 (**C**, CRT, 1 μM). RNA was extracted and taken for quantitative PCR analysis of mEpo and mL28 RNA. ΔΔCt values were calculated as a fold increase compared with the untreated control and means ± S.D. (*n* = 3); *** *p* < 0.001 was considered statistically significant compared with the control samples; ^##^ *p* < 0.01, ^###^ *p* < 0.001 compared with the S1P-stimulated samples.

In a next step, we investigated the mechanism of S1P-stimulated Epo production. To identify the S1P receptor subtype involved, we used selective S1P receptor antagonists, including NIBR-0213 for S1P_1_ [59], JTE-013 for S1P_2_ [60], and TY52156 for S1P_3_ [61]. S1P-stimulated Epo mRNA expression was completely reduced by NIBR-0213 and TY52156 (Figure 4A), but not by JTE-013, suggesting the involvement of both S1P_1_ and S1P_3_. Furthermore, the inhibitor of the classical ERK pathway (U0126) and an inhibitor of the stress-activated p38-MAPK (SB203580) both blocked the S1P effect (Figure 4B). As PKC was previously shown to be involved in hypoxia-induced Epo production in liver HepG2 cells [62,63,64,65], we tested the specific inhibitor of the Ca^2+^-dependent PKC (PKCα), CGP41251 (later renamed PKC412) [66], the pan-PKC inhibitor RO-318220 [67], and the PKD inhibitor CRT0066101 [68]. Notably, PKD was originally identified as a PKC isoform, named PKCμ, but it is now clear that PKD, again consisting of three isoforms, is activated by PKCs, thus building a PKC/PKD signaling axis [69]. The S1P effect on Epo mRNA was reduced by all PKC and PKD inhibitors. The fact that the PKCα inhibitor CGP41251 blocked the S1P effect at nM concentrations suggests that this isoform is involved in the S1P-mediated Epo production, possibly acting through PKD.

**Figure 5:**
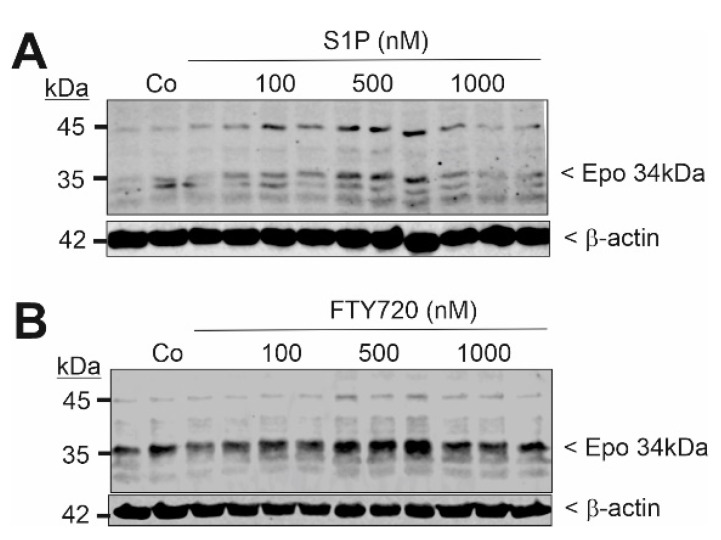
Effect of S1P and FTY720 on Epo protein expression in the human neuroblastoma cell line Kelly. Confluent Kelly cells were incubated for 16 h with serum-free DMEM prior to stimulation for 6 h with either vehicle (Co) or the indicated concentrations of S1P (**A**) and FTY720 (**B**). Thereafter, protein extracts were prepared and taken for protein separation by SDS-PAGE, transferred to nitrocellulose membranes, and subjected to Western blot analyses using antibodies against Epo and β-actin. Blots show one representative experiment performed in triplicates. Bands corresponding to 34 kDa Epo and β-actin were evaluated by Image Studio Lite software and the results are depicted in Supplementary Figure S4.

To investigate whether the observed effect of S1P on Epo production in F3-5 cells is of a more general nature, we also studied the neuroblastoma cell line Kelly, which is well-known to produce Epo under hypoxic condition in culture [8,23]. Cells were stimulated for 6 h with increasing concentrations of S1P and FTY720. Both compounds were able to upregulate 34 kDa Epo protein expression (Figure 5), thus confirming a general regulatory mechanism of Epo induction by S1P.

## 3. Discussion

In this study, we demonstrate for the first time that S1P is able to stimulate Epo protein synthesis and secretion in renal fibroblast-like cells in culture even under normoxic conditions, suggesting that S1P may contribute to erythropoiesis. So far, the best-characterized stimulus for renal Epo production is the hypoxia-induced stabilization of the transcription factor HIF-2α, which then binds to HREs in the Epo promoter and enhancer regions and activates Epo transcription and de novo protein synthesis [57,70].

Of interest, S1P was previously shown to activate HIF-1α and Epo production in mouse macrophages. S1P was found to be released from various immune cells when undergoing apoptosis and then acted through S1PRs on macrophages to synthesize Epo, which occurs in parallel to an upregulation of the EPOR [71]. These two events then led to a second loop of signaling, where secreted Epo acted on the EPOR and subsequently induced PPARγ to promote phagocytosis of dying cells by macrophages. Additionally, Epo suppressed the synthesis of pro-inflammatory cytokines and increased the synthesis of anti-inflammatory factors in macrophages, thereby mediating immune tolerance in vivo, as shown in a lupus nephritis model [71]. Apparently, in macrophages, HIF-1α is responsible for Epo production, which contrasts to Epo production in the kidney, which exclusively depends on HIF-2α. Moreover, it must be stressed that, in humans, macrophage-derived Epo plays no substantial role in erythropoiesis. For this function, Epo production of the kidney is mandatory and a loss of kidney function, which leads to loss of Epo production and anemia, can be compensated to a minor extent only by the liver, but not at all by macrophages [8,22,72].

In our study, in renal fibroblast F3-5 cells, S1P-stimulated Epo production occurs through activation of S1P_1_ and S1P_3_ receptors expressed on F3-5 cells. Within the signal transduction pathway of S1P, we identified PKC and PKD, p38-MAPK, and ERK as essential protein kinases. Blocking either of these kinases strongly suppressed S1P-stimulated Epo mRNA expression. In this view, it is known that HIF-1α stability is regulated by various post-translational modifications, such as hydroxylation, ubiquitination, SUMOylation, acetylation, methylation, and phosphorylation. Notably, phosphorylation by various protein kinases, including ERK, p38-MAPK, CDK1, Plk3, and GSK3β, occurs at several sites and can either promote HIF-1α stability [73,74,75] or degradation [76,77]. PKCs, especially the α and δ isoenzymes, have also been reported to stimulate HIF-1α transcriptional activity [78,79], although this may occur indirectly through ERKs or PKD. The same holds true for HIF-2α, where multiple post-translational modifications have been described. In view of several previous studies on the regulation of hypoxia-stimulated Epo production in hepatoma cells, PKC can be expected to regulate HIF-2α as well. In this context, in HepG2 cells, PKCα has a permissive effect in Epo production, as the inhibitors of Ca^2+^-dependent PKCs, i.e., CGP41251 and staurosporine, and downregulation of PKCα by prolonged phorbol ester treatment, reduced Epo production [62,65]. These data agree with our data in F3-5 cells, showing that CGP41251 and the pan-PKC inhibitor RO-318220 blocked S1P-stimulated Epo production (Figure 4C), thus confirming the role of PKCα.

It is presently not fully understood what is the role of the three HIF-α subtypes, i.e., HIF-1α, -2α, and -3α. As HIF-1α and HIF-2α staining of kidney sections from rats exposed to systemic hypoxia revealed no overlap between the two subtypes, and owing to the finding that peritubular fibroblasts stained exclusively positive for HIF-2α, it was concluded that HIF-2α is the main subtype stimulating Epo production [55,57]. In addition, knockdown of HIF-2α, but not HIF-1α, reduced Epo production [80,81]. Nevertheless, both subtypes are expressed in cultures of isolated renal fibroblasts, and they show a differential stabilization kinetics towards hypoxia [52]. Although HIF-1α and HIF-2α share many target genes, there is a subset of genes that are uniquely regulated by HIF-2α, such as Epo and genes involved in iron metabolism, while others are uniquely regulated by HIF-1α, including the glucose transporter 1 (GLUT1) and glycolytic enzymes [23,82].

Interestingly, Bouquerel et al. recently reported that, in several cancer cell types, including renal cancer cells, hypoxia led to increased S1P production and secretion and autocrine action through S1PRs to stabilize HIF-2α, and thereby to drive a more aggressive cancer phenotype [51]. They showed that this mechanistically involved phospholipase D activation, although the detailed signalling was not resolved. The same authors further showed that FTY720 abolished HIF-1α and HIF-2α protein expression in renal carcinoma cells by downregulating S1P_1_ [83]. In another study, Hait et al. showed that Sphk2 and S1P directly interact with the PAS domain of HIF-1α in the nucleus and promote its transcriptional activity. Depletion of Sphk2 by siRNA downregulated not only HIF-1α, but also HIF-2α mRNA and protein [84]. Although such a direct interaction of nuclear S1P with a transcription factor has been proposed before for histone deacetylases (HDACs) [85], this mechanism is unlikely to occur in F3-5 cells stimulated with extracellular S1P.

In both F3-5 and Kelly cells, the therapeutic drug FTY720 (fingolimod) increased Epo production. FTY720 primarily acts as an unselective S1PR agonist, activating all S1PR subtypes except S1P_2_. Therefore, the effect on Epo production is consistent with its agonistic effect on S1P_1_ and S1P_3_. However, in long-term treatment situations, FTY720 exerts its therapeutic effect on lymphocyte depletion by a functional antagonistic effect on S1P_1_, which derives from a sustained internalization and degradation of S1P_1_. Therefore, in a long-term therapeutic setting with fingolimod, as occurs in therapy of multiple sclerosis, no increased Epo is expected. Consistently, in patients treated with fingolimod, no polycythemia is reported. Notably, in vitro incubation of erythrocytes with a high concentration of fingolimod (10 μM) rather induced eryptosis [86].

Recently, a novel S1PR modulator, SAR247799, has been described that has an endothelial barrier protective capacity without causing lymphopenia. This compound is a G protein-biased selective S1P_1_ agonist that activates the G_i/0_ pathway more potently than the β-arrestin recruitment and S1P_1_ internalization [87]. Therefore, the compound will activate S1P_1_ without receptor desensitization and lymphocyte depletion [87,88]. Regarding renal Epo production, it would be very interesting to see whether this compound can stimulate Epo production in a more long-term situation.

**Figure 6:**
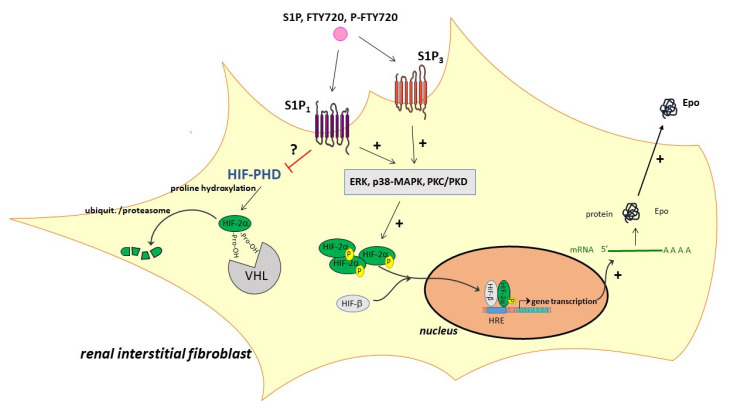
Schematic summary of the signal transduction pathways involved from S1P to Epo synthesis in interstitial fibroblasts. Epo, erythropoietin; ERK, extracellular signal-regulated protein kinases; PKC, protein kinase C; PKD, protein kinase D; HIF, hypoxia-inducible factor; HIF-PHD, HIF prolyl hydroxylase; HRE, hypoxia response element; VHL, von Hippel–Lindau protein.

Altogether, our data showed that, in interstitial fibroblasts, activation of S1P_1_ and S1P_3_ can trigger Epo production (Figure 6), and this could have a therapeutic impact for future treatment strategies of anemia in patients with CKD, or where a tissue-protective effect of Epo is desirable to prevent organ injury during acute and chronic inflammatory disease.

## 4. Materials and Methods

### 4.1. Chemicals

All chemicals, primer sequences, and antibodies are indicated in the Supplementary Data file.

### 4.2. Cell Lines and Cell Culture Conditions

The mouse fibroblast-like cell line F3-5, obtained by single-cell cloning from kidneys of transgenic Epo-Cre^ERT2^ mice, was isolated and characterized as previously described [52]. F3-5 cells were cultured in Dulbecco’s modified Eagle’s medium (DMEM) supplemented with 10% (*v*/*v*) fetal bovine serum (FBS), 10 mM HEPES pH 7.4, 100 units/mL penicillin, and 100 µg/mL streptomycin. The human neuroblastoma cell line Kelly was obtained from the European Collection of Authenticated Cell Cultures (ECACC) through Sigma Aldrich. Kelly cells were cultivated in RPMI medium supplemented with 10% (*v*/*v*) fetal bovine serum (FBS), 10 mM HEPES pH 7.4, 100 units/mL penicillin, and 100 µg/mL streptomycin. All cells were grown at 37 °C in a humidified atmosphere containing 5% CO_2_. Prior to stimulation, cells were rendered serum-free for 16 h in DMEM containing 10 mM HEPES and 0.1 mg/mL fatty acid-free bovine serum albumin (BSA).

### 4.3. Cell Stimulation, Homogenization, and Western Blotting

For hypoxia exposures, a hypoxia chamber (Whitley H35 HEPA Hypoxystation; Don Whitley Scientific) was used at 1% oxygen. Stimulated cells were washed with ice-cold phosphate buffered saline solution and were subsequently scraped with lysis buffer (50 mM Tris-HCl pH 7.4, 150 mM NaCl, 10% glycerol, 1% Triton X100, 2 mM EDTA pH 8.4, 2 mM EGTA pH 8.0, 40 mM β-glycerol phosphate, 50 mM sodium fluorid, 10 mM sodium pyrophosphate, 2 mM dithiothreitol, 200 µM sodium vanadate, 400 µL reconstituted Complete™ protease inhibitor cocktail, and 10 µM phenylmethylsulfonylfluoride). Cells were homogenized by sonication (5 s at 30 microns peak to peak amplitude; n. Zivy & Co Ltd., Oberwil, Switzerland), lysates were centrifuged for 10 min at 14,000× *g*, and the supernatant was taken for protein determination according to Bradford. For secreted Epo detection, cell supernatant after stimulation was taken for protein precipitation. Therefore, 5 mL of supernatant was incubated with trichloroacetic acid (final conc. 7% *(v*/*v*)) for 30 min on ice. Samples were then centrifuged for 30 min at 14,000× *g* at 4 °C. The precipitated proteins were further processed similar to the cell lysates, i.e., dissolved in Laemmli buffer and separated by SDS-PAGE followed by protein transfer to nitrocellulose membranes using a semi-dry blotter (Trans-blot Turbo^TM^ by Bio-Rad, Laboratories AG, Cressier, Switzerland). Membranes were blocked with 3% (w/v) low-fat milk powder in PBS for 1 h and were then incubated for 16 h at 4 °C with the respective antibodies diluted in a buffer containing 50 mM Tris-HCl pH 7.4, 200 mM NaCl, 10% (*v*/*v*) horse serum, 3% (w/v) BSA fraction V, and 0.1% (*v*/*v*) Tween 20. Secondary fluorescent-tagged antibodies were from LI-COR Biosciences (Bad Homburg, Germany), and development was done in a Licor fluorescence-chemiluminescence detector. Bands were evaluated using the Image Studio Lite software (LI-COR Biosciences, Bad Homburg, Germany). All antibodies and their dilutions are listed in the Supplementary Materials. 

### 4.4. RNA Extraction and Quantitative Real-Time PCR Analysis

Stimulated cells were washed with ice-cold PBS and homogenized in RNA-Solv reagent. Total RNA extraction was performed according to the instructions of the manufacturer. The yield and purity of the isolates were assessed with a NanoDrop ND-1000 spectrophotometer (Witec AG, Littau, Switzerland). First strand cDNA was synthesized using 2 µg of total RNA as template. SYBR Green-based quantitative PCR was performed in a BioRad CFX Connect™ Optics Module thermal cycler (Bio-Rad Laboratories Inc., Hercules, CA, USA). The Bio-Rad CFX Manager software was used to monitor the melting curve and to obtain the quantification data. The relative mRNA expression of the gene of interest was calculated with the ∆∆Ct method normalized to L28 mRNA as housekeeping gene. The following primers were used: mEpo: forward: AAT GGA GGT GGA AGA ACA GG, reverse: ACC CGA AGC AGT GAA GTG A; mL28 forward: GCA AAG GGG TCG TGG TAG TT, reverse: TTC TGG CTT CGA AGG ATG GC.

### 4.5. Statistical Analysis

Statistical analysis was performed by one-way ANOVA or an unpaired t-test, where applicable. For multiple comparisons, the level of significance was calculated with Bonferroni correction. GraphPad Prism Software, version 8.4.2. (San Diego, CA, USA) was used for statistical analysis presentations. 

## Data Availability

The authors declare that all data supporting the findings of this study are available within this paper or within the supplementary file, or can be obtained from the corresponding author up on request.

## Supplementary Materials

The following are available online at https://www.mdpi.com/article/10.3390/ijms22179467/s1.

## Abbreviations

βcR.	β-common receptor
BSA	bovine serum albumin
CKD	chronic kidney disease
DMEM	Dulbecco’s modified Eagle medium
Epo	erythropoietin
FBS	fetal bovine serum
GPCR	G protein-coupled receptor
HIF	hypoxia-inducible factor
HRE	hypoxia response element
kd	knockdown
PBS	phosphate-buffered saline
PI3K	phosphoinositide 3-kinase
PK	protein kinase
REPC	renal Epo producing cells
S1P	sphingosine 1-phosphate
SDS-PAGE	sodium dodecyl sulfate-polyacrylamide gel electrophoresis
shRNA	small hairpin RNA
SphK	sphingosine kinase
UUO	unilateral ureteral obstruction
