# Supplementary material for: S1P Stimulates Erythropoietin Production in Mouse Renal Interstitial Fibroblasts by S1P1 and S1P3 Receptor Activation and HIF-2α Stabilization
Source: Int J Mol Sci. 2021 Aug 31;22(17):9467. doi: 10.3390/ijms22179467 (PMC8430949; doi:10.3390/ijms22179467)
Supplement: Supplementary file 1 [file ijms-22-09467-s001.zip › ijms-1351754-Supplementary.pdf]

Supplementary Methods and Data

Hafizi et al.: S1P stimulates erythropoietin production in mouse renal interstitial fibroblasts by $S1P_1$ and $S1P_3$ receptor activation and HIF-2α stabilization.

Chemicals:

S1P was from Avanti Polar Lipids Inc. (Alabaster, AL, USA); FTY720, FTY720-P, CRT0066101, phosphate buffered saline (PBS), Kapa SYBR FAST, protease inhibitor cocktail, fatty acid-free bovine serum albumin (BSA), BSA fraction V, puromycin, dimethyl sulfoxide (DMSO), and horse serum were from Sigma-Aldrich Chemie GmbH (Buchs, Switzerland). NIBR-0213 was from GLixx Laboratories (Hopkinton, MA, USA); JTE-013 and TY52156 were from Tocris Bioscience (Bristol, UK); RO-318220 and U0126 were from Labforce AG (Muttenz, Switzerland); CGP41251 (PKC412) and SB203580 were from Selleckchem (Houston, TX, USA); ponesimod was from Cayman Chemical (Ann Arbor, Michigan, USA); compound 2 was purchased from Axon Medchem (Groningen, Netherlands). RNA-Solv® reagent from Omega Bio-tek Inc., (Norcross, GA, USA); First Strand cDNA Synthesis kit was from Thermo Scientific (Waltham, MA, USA). IRDye® 800CW secondary antibodies were from LI-COR Biosciences (Lincoln, NE, USA). Trypsin-EDTA 0.25%, DMEM containing Glutamax™, pyruvate and 4.5 g/L D-glucose were from Gibco® by Life Technologies Limited (Paisley, UK). Fetal bovine serum (FBS) was purchased from PANBiotech GmbH (Catalogue No. P40-37, Aidenbach, Germany). Transblot Turbo RTA transfer kit was from BioRad Laboratories (Hercules, CA, USA). All oligonucleotide primers were from Eurofins Genomics GmbH (Ebersberg, Germany). Standard chemicals were of highest possible grade and either from Carl Roth GmbH (Karlsruhe, Germany) or from Sigma-Aldrich Chemie GmbH (Buchs, Switzerland).

Antibodies used:

Commercially available antibodies used were: Epo (St. John's Laboratory STJ27630, 1:1000), HIF-2α (Bethyl Laboratories A700-003, 1:1000), β-actin (Sigma, A-1978 1:5000), α-tubulin (Sigma, T9026 1:3000). IRDye® 800CW secondary antibodies were from LI-COR Biosciences (Lincoln, NE, USA).

Supplementary Figures:

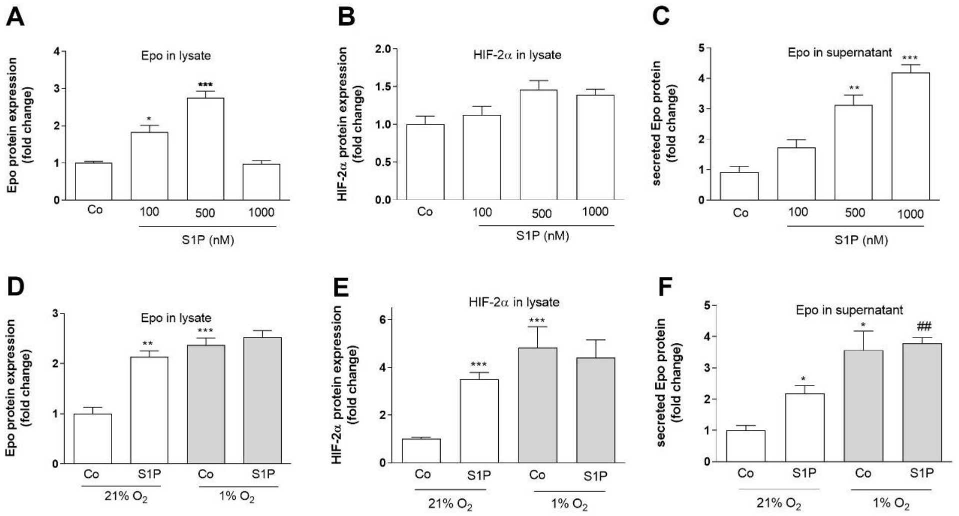

Figure S1: Effect of S1P and hypoxia on protein expression of Epo and HIF-2α in the immortalized mouse renal fibroblast cell line FAIK F3-5. Blots shown in Figure. 1 were evaluated by Image Studio Lite software and protein expression of 34 kDa Epo and 118 kDa HIF-2α were normalized to β-actin. Results are depicted as fold change and are means ± S.D. (*n*=3), * $p < 0.05$, ** $p < 0.01$, *** $p < 0.001$ compared to the normoxic-Co values; ## $p < 0.01$ compared to the normoxic-S1P values.

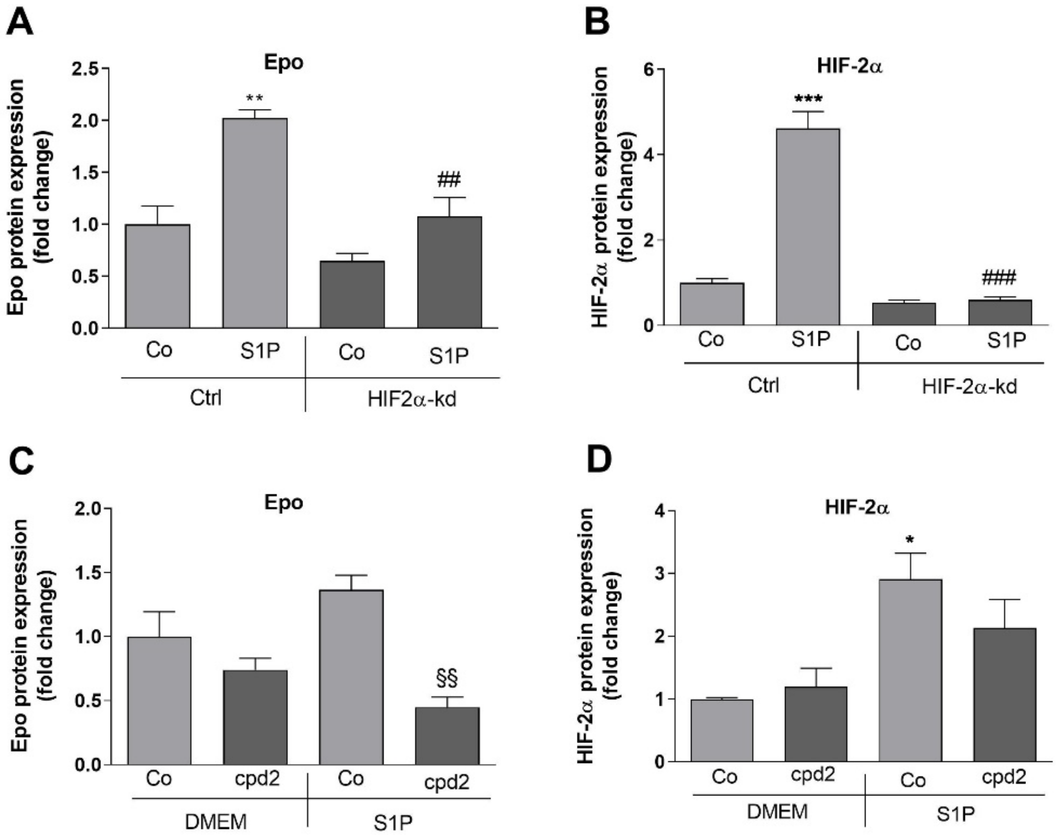

Figure S2: Effect of HIF-2α knockdown and a HIF-2α inhibitor on S1P-stimulated Epo protein expression in F3-5 cells. Blots shown in Figure. 2 were evaluated by Image Studio Lite software and the protein expression of 34 kDa Epo and *118 kDa* HIF-2α normalized to β-actin. Results are depicted as fold change and are means ± S.D. (n=3), * $p < 0.05$, ** $p < 0.01$, *** $p < 0.001$ compared to the Ctrl-Co or DMEM-Co values; ## $p < 0.01$, ### $p <$ 0.001 compared to the Ctrl-S1P values; §§ $p < 0.01$ compared to the S1P-Co values.

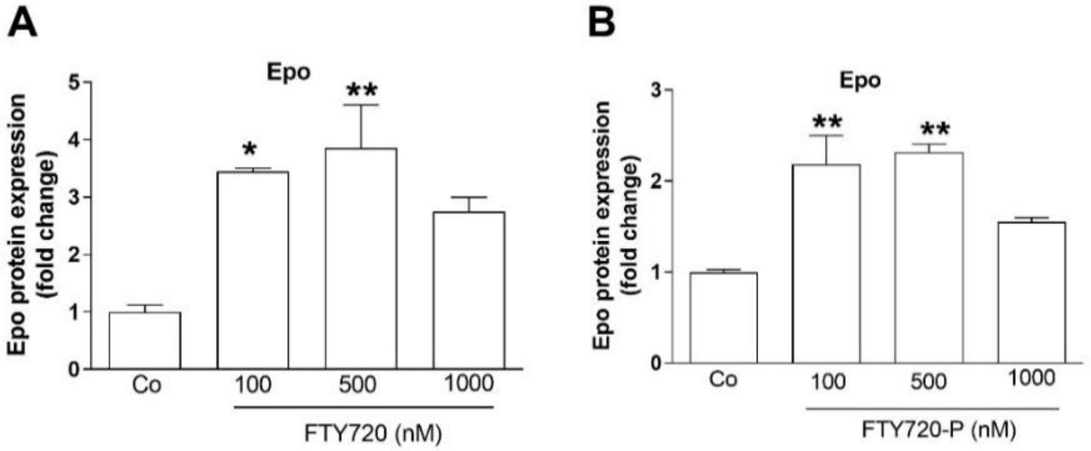

Figure S3: Effect of FTY720 and FTY720-phosphate on mouse Epo protein expression in F3-5 cells. Blots shown in Figure. 3 were evaluated by ImageStudioLite software and the protein expression of 34 kDa Epo was normalized to β-actin. Results are depicted as fold change and are means ± S.D. (n=3), * $p < 0.05$, ** $p < 0.01$ compared to the control values.

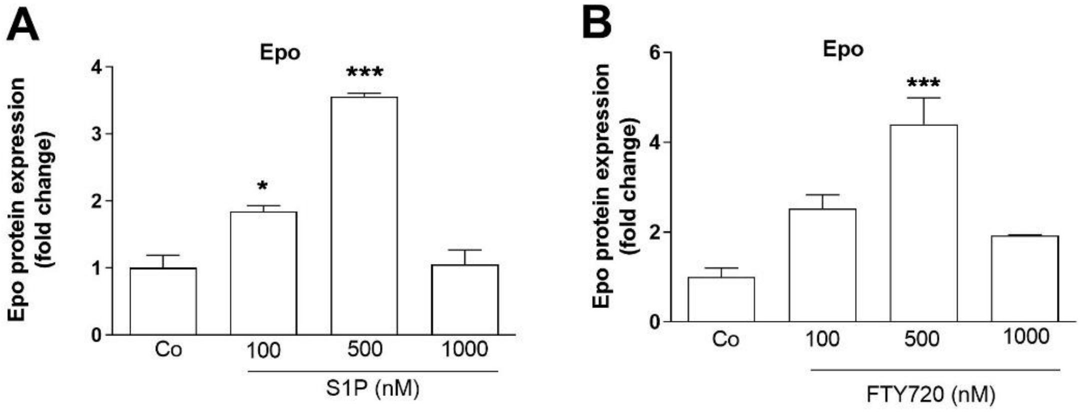

Figure S4: Effect of S1P and FTY720 on Epo protein expression in the human neuroblastoma cell line Kelly. Blots shown in Figure. 5 were evaluated by Image Studio Lite software and protein expression of 34 kDa Epo was normalized to β-actin. Results are depicted as fold change and are means ± S.D. (n=3), * p <0.05, *** p <0.001 compared to the control values.
